# Expression and characterisation of a *Sarcoptes scabiei* protein tyrosine kinase as a potential antigen for scabies diagnosis

**DOI:** 10.1038/s41598-017-10326-w

**Published:** 2017-08-29

**Authors:** Nengxing Shen, Ran He, Yuqing Liang, Jing Xu, Manli He, Yongjun Ren, Xiaobin Gu, Weimin Lai, Yue Xie, Xuerong Peng, Guangyou Yang

**Affiliations:** 1 Department of Parasitology, College of Veterinary Medicine, Sichuan Agricultural University, Wenjiang, 611130 China; 2 Sichuan Animal Sciences Academy, Sichuan Chengdu, 610066 China; 3 Animal Breeding and Genetics key Laboratory of Sichuan Province, Sichuan Chengdu, 610066 China; 4 Department of Chemistry, College of Life and Basic Science, Sichuan Agricultural University, Wenjiang, 611130 China

## Abstract

Scabies is a disease that harms humans and other animals that is caused by the itch mite *Sarcoptes scabiei* burrowing into the stratum corneum of the skin. In the early stages of scabies, symptoms are often subclinical and there are no effective diagnostic methods. Herein, we cloned, expressed and characterised an *S. scabiei* protein tyrosine kinase (SsPTK) and evaluated its diagnostic value as a recombinant antigen in rabbit during the early stages of *Sarcoptes* infestation. The SsPTK protein is ~30 kDa, lacks a signal peptide, and shares high homology with a PTK from the rabbit ear mite *Psoroptes ovis cuniculi*. The protein was widely distributed at the front end of mites, particularly in the chewing mouthparts and legs. Indirect ELISA using recombinant SsPTK showed good diagnostic value, with 95.2% (40/42) sensitivity and 94.1% (48/51) specificity for detecting anti-PTK antibody in serum samples from naturally-infested rabbits. More importantly, PTK ELISA could diagnose infection in the early stages (infestation for 1 week) with an accuracy of 100% (24/24). SsPTK therefore shows potential as a sensitive antigen for the early diagnosis of parasitic mite infestation.

## Introduction

Scabies is a highly contagious parasitic disease threatening human and animal health^1^. *Sarcoptes scabiei*, the etiological agent, causes symptoms including skin inflammation, itching and crusty lesions on the skin^2^, triggering a series of health problems, particularly in socially vulnerable groups such as indigenous populations^3^ and people in impoverished areas of some developing countries^4^. Worldwide, ~300 million people are infested with scabies mites every year^5, 6^, and more than 100 species of animals suffer from *S. scabiei* infestation^7^, resulting in serious health problems and economic losses. Secondary bacterial infestation occurs in many cases when skin lesions are not treated in a timely manner^8, 9^. Scabies eradication is an arduous task in developing countries^10^ due to poverty and poor living conditions^11^. Improvements in health education, sanitary conditions and treatment of scabies are frequently impractical in developing countries^9^.

Functional genes, allergy antigens and vaccines for *S. Scabiei* are currently being explored for the prevention and treatment of scabies^12–18^. For instance, inactivated serine proteases have been linked to immune evasion in scabies mites^19^, and an aspartic protease plays a role in the digestion of host skin and serum molecules, and could be a target for acaricidal drugs that lower mite survival^20^. Moreover, recombinant *S. scabiei* apolipoprotein antigen Sar s 14.3 is a promising immunodiagnostic that elicits high levels of IgE and IgG^21^. However, during the early stages of infestation, when symptoms are generally subclinical, one cannot easily distinguish scabies from lice, crab lice, eczema and hairless tinea. To date, no effective medical diagnostic technology is available for confirmatory diagnosis of early scabies. Thus, it is imperative that effective diagnostic methods are developed. Specific serum antibodies can be detected in the early stages of infestation and represent promising targets for use in immune diagnostic methods such as enzyme-linked immunosorbent assays (ELISA)^22^. Importantly, several studies have shown that crude *S. scabiei* proteins can be used to detect mite infestation by serological ELISA with varying levels of sensitivity and specificity^23–26^. Additionally, recombinant proteins have also been employed in preliminary dot-ELISA and indirect-ELISA approaches for diagnosis of scabies^27, 28^. However, these methods appear to suffer from insufficient sensitivity and/or specificity for detection during the early stages of the disease.

Protein tyrosine kinases (PTKs) play an influential role in intracellular signal transduction and regulate various cellular functions such as metabolism, segmentation, differentiation and cell death^29^. PTKs are also critical in regulation of the adaptive immune response^30^. For example, during phosphorylation of tyrosines, PTKS, coupled with Syk or the related ZAP-70 tyrosine kinase, mediate recruitment and activation^30^. Importantly, PTK expression can alter the development of the immune response during the recruitment and activation process when mites attack hosts. Given the importance of PTKs to parasite biology and immunology, they may be promising antigen candidates for serological detection, but their potential in *S. scabiei* has not been explored.

In the present study, we report the cloning, expression and purification of a PTK from *S. scabiei*. Fluorescent immunolocalisation was performed to determine the distribution in mite tissues. We also evaluated the potential of recombinant SsPTK as a diagnostic antigen for the early diagnosis of rabbit mite infestation by ELISA.

## Results

### Sequence analysis of PTK

**Figure 1:**
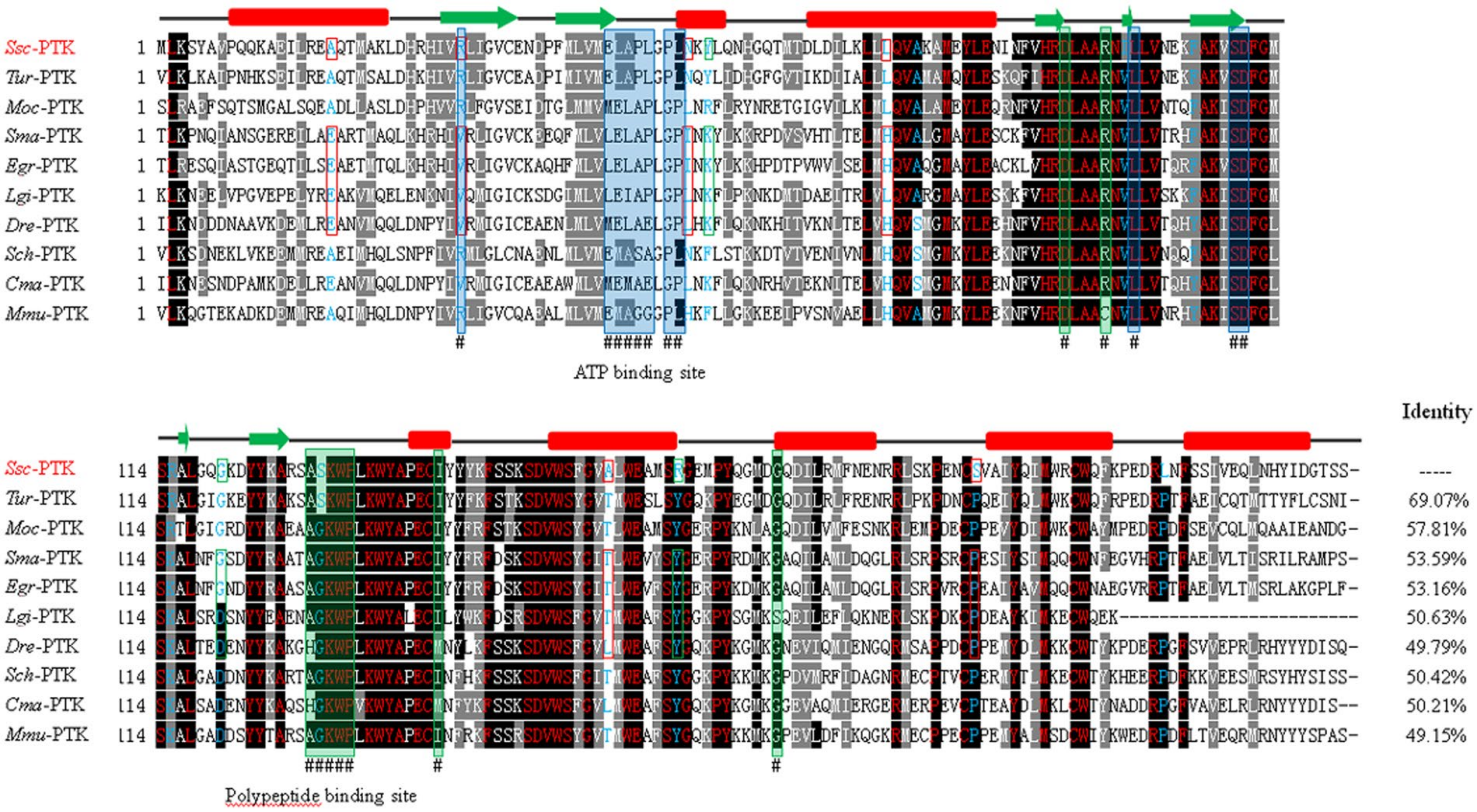
Sequence alignment of *S. scabiei* PTK with PTK proteins from other species. Alignment of the deduced amino acid sequence of SsPTK (*Sarcoptes scabiei*, KY080515) is shown with homologs from other species. The percentage homology between SsPTK and each of the other PTKs is shown at the end of the alignment. Active site residues that are the same or different between SsPTK and other PTKs are indicated by red and blue letters, respectively. A change in charge is marked with green boxes (*S. scabiei*-mammalian host, Tyr57-Arg/Lys57, Gly120-Asp120 and Arg166-Tyr166). Exchanges of polar and non-polar residues (Ala18-Glu18, Arg31-Val31 and Ser196-Pro196) are marked with red boxes. The ATP binding site is highlighted by a blue background, and the polypeptide binding site with a green background. The predicted secondary structure of SsPTK is displayed above the alignment. Accession numbers are as follows: *Ssc*-PTK (*Sarcoptes scabiei*, KY080515); *Tur*-PTK (*Tetranychus urticae*, XP_015789937.1); *Moc*-PTK (*Metaseiulus occidentalis*, XP_003748017.1); *Sma*-PTK (*Schistosoma mansoni*, CCD80719.1); *Egr*-PTK (*Echinococcus granulosus*, CDS21451.1); *Lgi*-PTK (*Lottia gigantean*, XP_009054879.1); *Dre*-PTK (*Danio rerio*, AAK49118.1); *Sch*-PTK (*Siniperca chuatsi*, AKA66303.1); *Cma*-PTK (*Chlamydotis macqueenii*, KFP38041.1); *Mmu*-PTK (*Mus musculus*, AAB36538.1).

**Figure 2:**
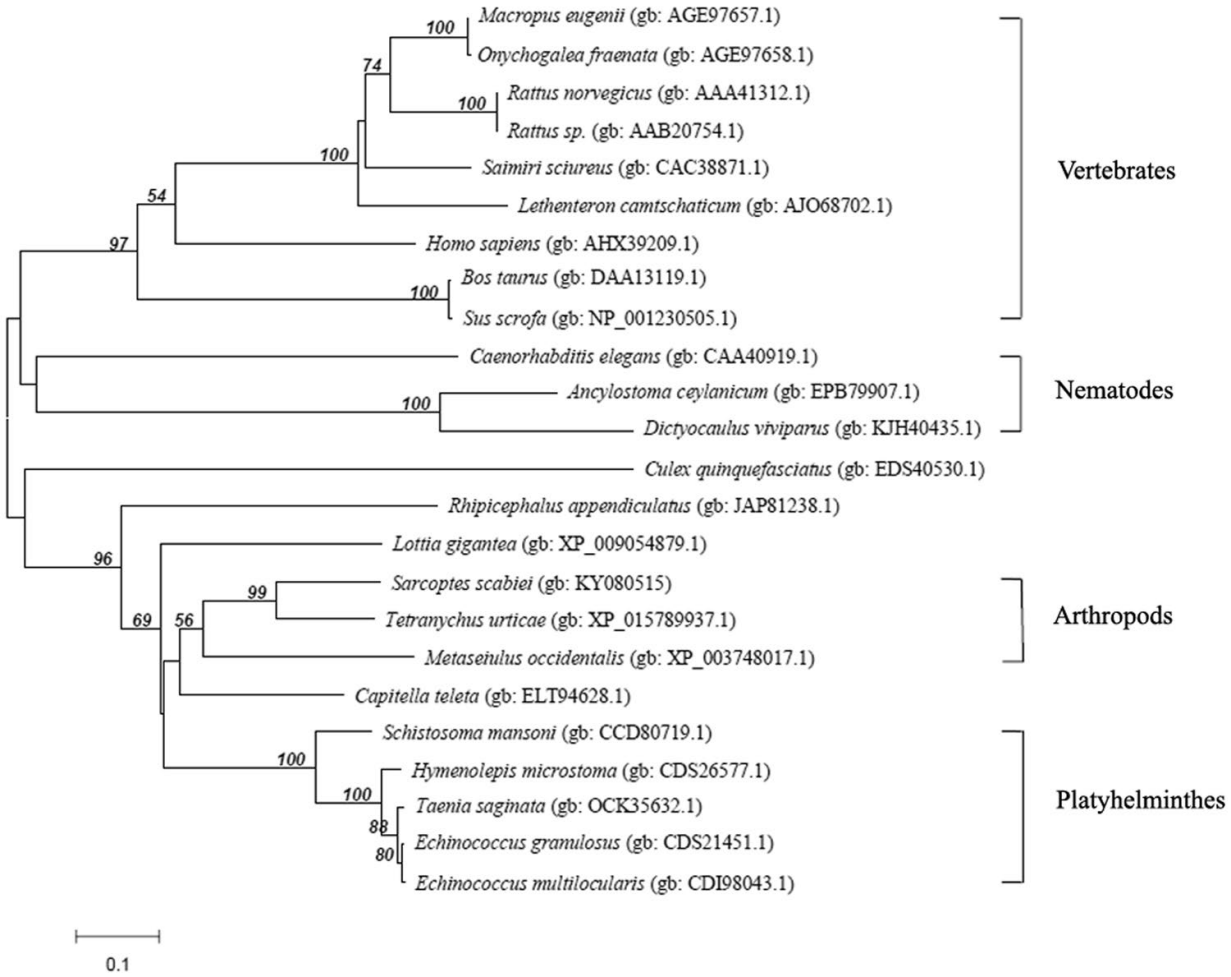
Phylogenetic relationships between SsPTK and other PTKs. The unrooted phylogenetic tree was constructed using PTK sequences from 24 species by the neighbour-joining (NJ) method in MEGA software. PTK sequences used in the tree (with their GenBank or SwissProt accession numbers) are as follows: *Sarcoptes scabiei* (KY080515); *Macropus eugenii* (AGE97657.1); *Onychogalea fraenata* (AGE97658.1); *Rattus norvegicus* (AAA41312.1); *Rattus sp*. (AAB20754.1); *Saimiri sciureus* (CAC38871.1); *Lethenteron camtschaticum* (AJO68702.1); *Homo sapiens* (AHX39209.1); *Bos taurus* (DAA13119.1); *Sus scrofa* (NP_001230505.1); *Caenorhabditis elegans* (CAA40919.1); *Ancylostoma ceylanicum* (EPB79907.1); *Dictyocaulus viviparous* (KJH40435.1); *Culex quinquefasciatus* (EDS40530.1); *Rhipicephalus appendiculatus* (JAP81238.1); *Lottia gigantean* (XP_009054879.1); *Tetranychus urticae* (XP_015789937.1); *Metaseiulus occidentalis* (XP_003748017.1); *Capitella teleta* (ELT94628.1); *Schistosoma mansoni* (CCD80719.1); *Hymenolepis microstoma* (CDS26577.1); *Taenia saginata* (OCK35632.1); *Echinococcus granulosus* (CDS21451.1); *Echinococcus multilocularis* (CDI98043.1).

The *SsPTK* gene sequence includes a 825 bp open reading frame (ORF) encoding a putative protein of 274 amino acid residues (~31.1 kDa) with a pI of 8.56, without a signal peptide or transmembrane domains, and with low hydrophobicity. SsPTK shares highest identity with a PTK from *Psoroptes ovis cuniculi* (85.53%) followed by and enzyme from *Lottia gigantean* (50.63%) and an enzyme from *Schistosoma mansoni* (53.59%). Moreover, *Ss*PTK shares 69.07% identity with a PTK from *Tetranychus urticae* 57.81% identity with a protein from *Metaseiulus occidentalis*, but only 50% identity with proteins in platyhelminthes and vertebrates (Fig. 1). Phylogenetic analysis showed that PTKs from 24 species clustered into four main branches (vertebrates, nematodes, arthropods and platyhelminths), with high bootstrap values between members of the same branch. SsPTK is in the arthropod branch (Fig. 2).

### Cloning, expression and purification of SsPTK

**Figure 3:**
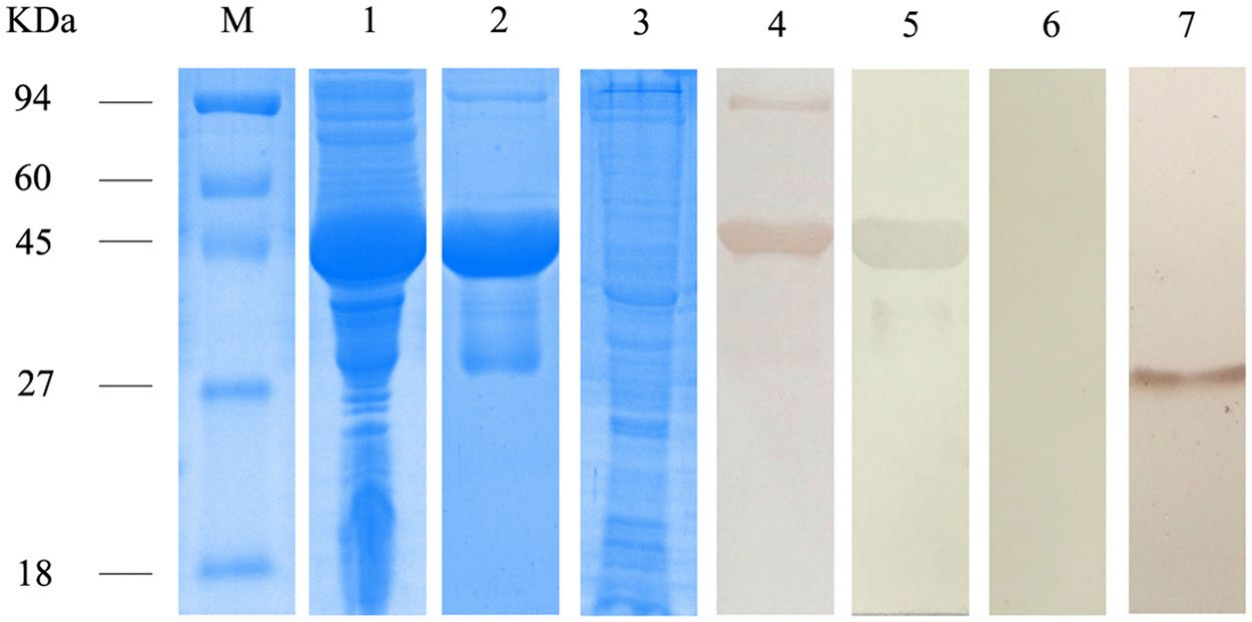
SDS-PAGE and western blotting of *S. scabiei* PTK. Lanes: M, protein molecular weight markers (in kDa); 1, non-purified recombinant PTK (inclusion bodies from *Escherichia* coli BL21 (DE3) expressing the protein); 2, purified recombinant PTK; 3, total crude proteins from *S. scabiei*; 4, purified recombinant SsPTK detected in serum (diluted 1:100 with 0.01 M PBS) from a rabbit naturally infested with *S. scabiei* (experimental group); 5, purified recombinant SsPTK detected in rabbit anti-PTK serum (diluted 1:100 with 0.01 M PBS; positive control); 6, purified recombinant SsPTK detected in naïve rabbit serum (diluted 1:100 with 0.01 M PBS; negative control); 7, total crude proteins detected with rabbit anti-PTK serum (diluted 1:100 with 0.01 M PBS). Samples derived from the same experiment and gels/blots were processed in parallel. Cropping was used and full-length blots/gels are presented in supplementary information Figure S1.

The 825 bp ORF sequence was successfully cloned and expressed in *E. coli* BL21 (DE3) cells. Expression of recombinant SsPTK was maximal after 8 h of induction with 1 mM IPTG (see supplementary Fig. S1-a) but the protein (~48 kDa including vector-encoded amino acids) was expressed in inclusion bodies (Fig. 3, lane 1). After purification and concentration, the recombinant protein and total crude protein from mites was assessed by 12% SDS-PAGE (Fig. 3, lanes 2 and 3).

### Western blot analysis

Serum samples from rabbits naturally infested with *S. scabiei* (experimental group) and rabbit anti-PTK serum (positive control) were used to detect the ~48 kDa purified recombinant SsPTK by western blotting. A strong reactivity and high antigenicity was observed (Fig. 3, lanes 4–5). No band was observed when naïve rabbit serum (negative control) was used (Fig. 3, lane 6). When total crude proteins were probed with anti-PTK rabbit serum, a ~30 kDa protein band was observed (Fig. 3, lane 7).

### Immunohistochemistry

**Figure 4:**
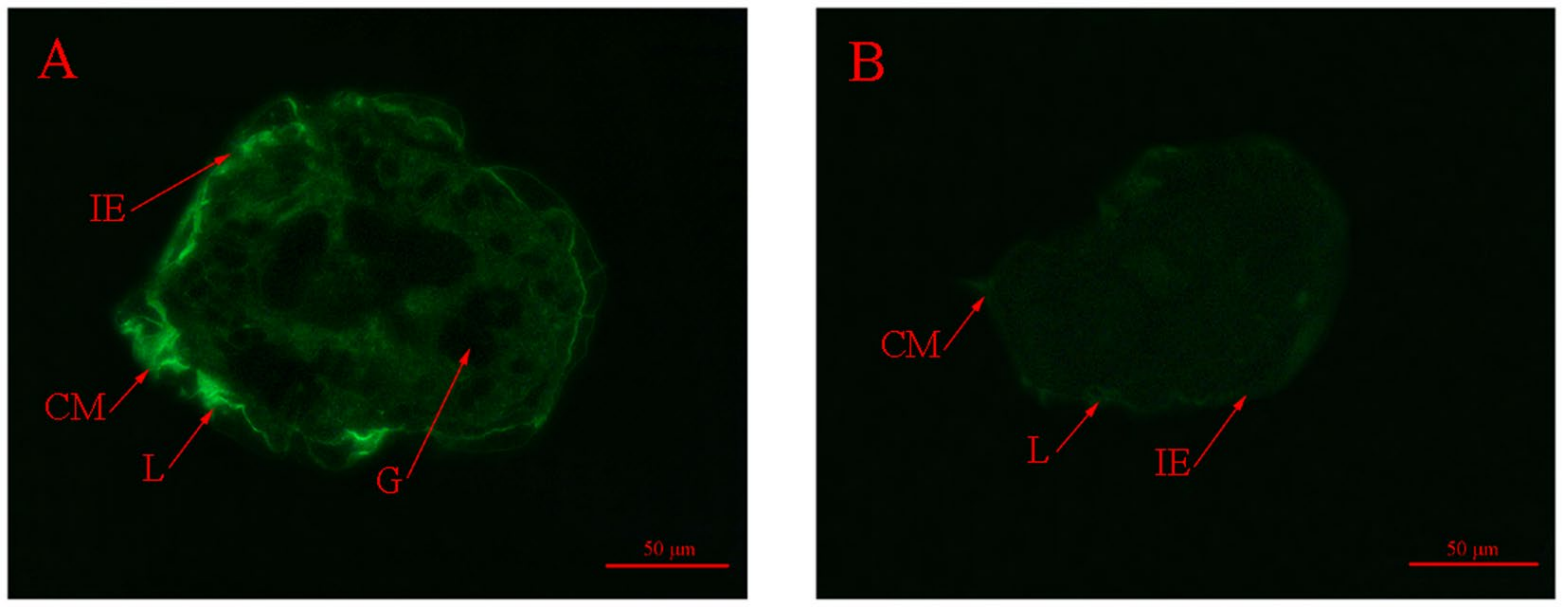
Immunolocalisation of PTK in *S. scabiei* tissue. (**A**) Staining with anti-PTK as the primary antibody; (**B**) negative control (no primary antibody). Annotation is as follows: IE, epidermal integument; CM, chewing mouthpart; L, leg; G, gut.

The distribution of SsPTK was determined by fluorescence localisation, and the protein was found to be widely distributed at the front end of mites, particularly in regions surrounding the chewing mouthparts and legs (Fig. 4A). No fluorescence signals were observed in the gut (Fig. 4A), or in the negative control samples (Fig. 4B).

### Establishment of indirect ELISA

**Figure 5:**
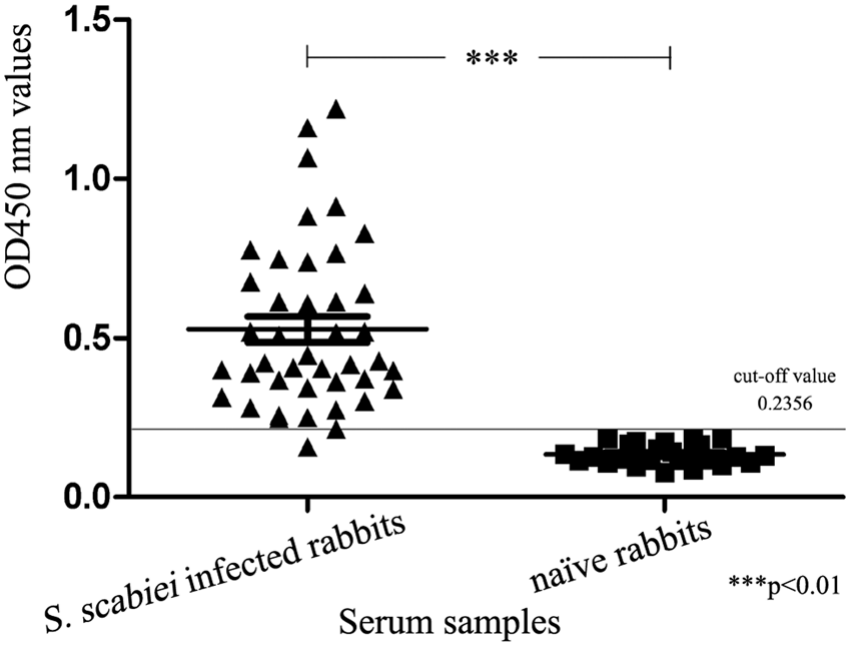
ELISA of serum samples from *S. scabiei*-infested rabbits and naïve rabbits. *S. scabiei* infested rabbits = OD_450_ values of serum samples (n = 42) from *S. scabiei*-infested rabbits; naïve rabbits = OD_450_ values of serum samples (n = 24) from naïve rabbits. *, ** and *** indicate statistical significance at *p* > 0.05, 0.01 < *p* < 0.05, and *p* < 0.01, respectively.

Based on a checkerboard titration study (see Methods), the optimal conditions for indirect ELISA were determined to be 4 μg/mL of recombinant SsPTK protein, a 1:80 serum dilution (see supplementary Table S1), and a 1:5000 dilution of secondary antibody. A total of 24 naïve rabbit sera were used to determine the OD_450_ cut-off value, which was 0.2356 (mean = 0.1351, SD = 0.0335). Therefore, an OD_450_ ≥ 0.2356 was determined as positive, and an OD_450_ < 0.2356 was determined as negative (Fig. 5).

### Sensitivity and specificity of the indirect ELISA

**Figure 6:**
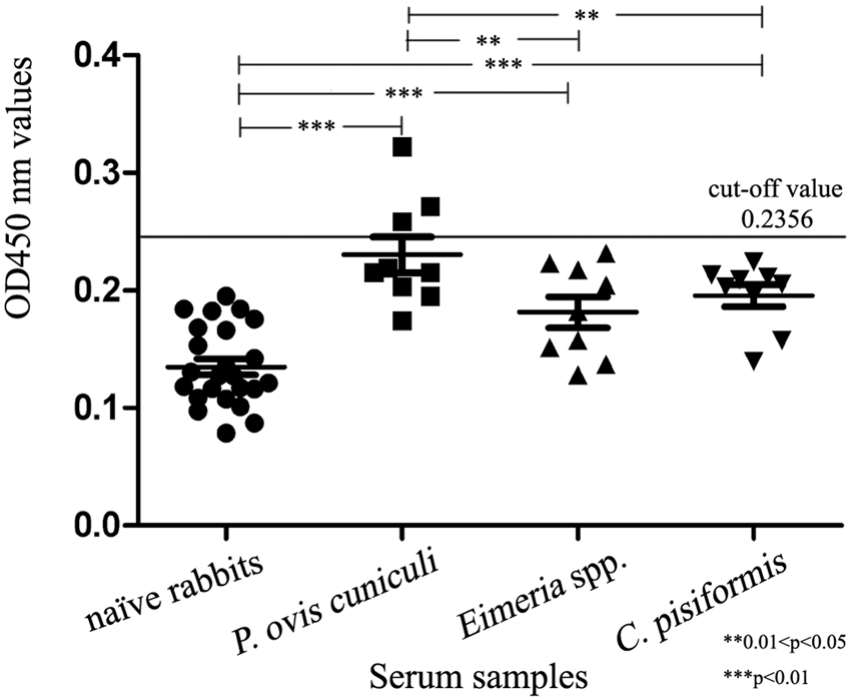
Cross-reactivity in ELISA. Naïve rabbits = OD_450_ values of serum samples (n = 24) from naïve rabbits; *P. ovis cuniculi* = OD_450_ values of serum samples (n = 9) from *P. ovis cuniculi*-infested rabbits; *Eimeria* spp. = OD_450_ values of serum samples (n = 9) from *Eimeria* spp.-infested rabbits; *C. pisiformis* = OD_450_ values of serum samples (n = 9) from *C. pisiformis*-infested rabbits. *, ** and *** indicate statistical significance at *p* > 0.05, 0.01 < *p* < 0.05 and *p* < 0.01, respectively.

Using the optimal conditions for indirect ELISA, detection of the specific IgG in serum samples was performed using samples from rabbits infested with *S. scabiei*, *Eimeria* spp., *P. ovis cuniculi* or *C. pisiformis*. The assay sensitivity was 95.2% (correct identification of 40 out of 42 parasitologically confirmed *S. scabiei* cases; Fig. 5). There was no cross-reactivity between the PTK antigen and serum from rabbits infested with *Eimeria* spp. or *C. pisiformis*, but cross-reactivity was observed with three of the nine serum samples from rabbits infested with *P. ovis cuniculi*. Thus, the specificity of the recombinant antigen based on indirect ELISA was 94.1% (48/51; Fig. 6).

### Repeatability and reproducibility of SsPTK ELISA

Coefficients of variation (CVs) for the intra-assay repeatability ranged from 2.80 to 5.96% (mean = 3.48%), while CVs for the inter-assay reproducibility ranged from 2.55 to 6.80% (mean = 3.77%). All CVs were <10%, suggesting the recombinant SsPTK-based ELISA was stable and reproducible.

### Use of SsPTK ELISA in early diagnosis

**Figure 7:**
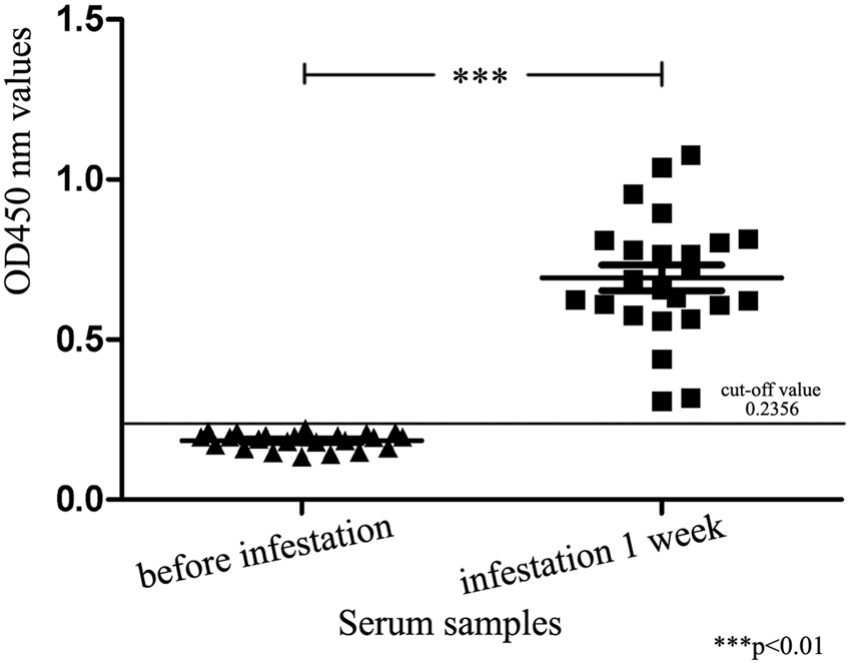
Early diagnosis testing of SsPTK ELISA. Before infestation = OD_450_ values of serum samples (n = 24) from rabbits before artificial infestation; Infestation 1 week = OD_450_ values of serum samples (n = 24) from rabbits after artificial infestation for 1 week. *, ** and *** indicate statistical significance at *p* > 0.05, 0.01 < *p* < 0.05 and *p* < 0.01, respectively.

Serum was collected from 24 rabbits before and after artificial infestation with mites for 1 and 2 weeks. The SsPTK ELISA method detected the anti-PTK IgG antibody in all 24 serum samples from rabbits artificially infested for 1 week (Fig. 7).

## Discussion

Diagnosis of scabies in humans and animals remains difficult, and no commercial immunodiagnostic tests are available for *S. scabiei* infestation. At present, diagnosis of mite infestation usually involves observation of clinical lesions and subsequent searching for the agent in infested skin for confirmation. However, only 45% of infested animals harbour mites in skin scrapings^26^, making accurate diagnosis difficult. A serodiagnostic approach using *S. scabiei* antigen-based ELISA would be an ideal alternative. The objective of this study was to utilise *S. scabiei* recombinant PTK to establish an ELISA method, evaluate its sensitivity and specificity, and test its diagnostic potential during the early stages of mite infestation.

The SsPTK gene was cloned and characterised. Phylogenetic tree analysis classified SsPTK into the arthropod branch with 99% bootstrap values between *Ss*PTK and PTK from *Tetranychus* urticae. SsPTK is also closely related PTK from other arthropods including *P. ovis cuniculi*, *Culex quinquefasciatus* and *Rhipicephalus appendiculatus*. Such close genetic relationships imply that arthropod PTKs perform similar functions. Amino acid sequence comparison revealed a highly conserved region between different species, suggesting PTK transfers the ATP gamma phosphate to protein tyrosine residues of various substrate proteins in these organisms. Mutation of this gene alter the function with physiologically important effects. The main function of the enzyme is the regulation of intracellular signal transduction, especially immune signal transduction^31^. Amino acid sequence analysis indicated that SsPTK may perform special functions, and mutation may lead to immune cell defects^32, 33^, making it a potential target for immunotherapy of mites.

Fluorescence immunohistochemistry assays revealed a strong fluorescence signal in the chewing mouthparts and legs of mites. Given that PTKs have a vital function in the immune response^30^, this suggests that the early immune response in *S. scabiei* is induced in these parts of the mite, which may be the main organs harbouring endogenic PTK. This finding expands our understanding of the development of the immune response following the attaching of mites on hosts, but further research is required.

Because *S. scabiei* infestation usually lacks obvious clinical symptoms, it is difficult to diagnose scabies from lice, crab lice, eczema and hairless tinea. The traditional diagnostic method, based on the observation of skin scrapings, is limited, inconvenient and ineffective, especially for the early diagnosis of scabies^23, 34, 35^. Furthermore, immunoscreening to identify antigenic proteins is onerous for *S. scabiei* ^36^. Given that there are no effective tools for harvesting crude allergen proteins, purified recombinant allergens are increasingly being used as serological diagnostic agents^37^. For instance, hydatid disease, coenurosis, nematodes and protozoa can be diagnosed using recombinant allergen-based methods^38, 39^. Additionally, the total IgG responses to *S. scabiei* can be observed in severely infested individuals. Building on this finding, we developed a PTK-based ELISA that performed well for the detection of *S. scabiei* infestation, with 95.2% sensitivity and 94.1% specificity, which was better than that achieved in our previous study using the recombinant cofilin protein (83.3% sensitivity, 87.9% specificity)^28^. Previous studies indicated that rSar s 14.3 can bind a specific IgE but only when high levels of IgE are found during severe disease cases^40, 41^. Thus, recombinant Sar s 14.3 is unsuitable for diagnosing scabies during the early phase of the pathogenetic process. In early stage tests in the present study (infestation for 1 week), all 24 positive serum samples were correctly diagnosed by SsPTK-based ELISA, and the performance was significantly higher with samples before artificial infestation (p < 0.01). This suggests the anti-PTK antibody was significantly increased in the early stages of mite infestation. Of note, when using serum samples from rabbits artificially infested for 2 weeks, we also detected the anti-PTK antibody, but the results were not significantly different from those of animals infested for 1 week (p > 0.05; data is not shown). Recombinant SsPTK therefore has potential serodiagnosic value for detection during the early stages of scabies.

Our SsPTK-based ELISA showed high sensitivity and specificity for diagnosis of mites in rabbits. The total IgG levels in 42 serum samples from severely infested rabbits were significantly higher than those in naïve rabbits (p < 0.01). After infestation, anti-PTK antibody levels were clearly altered in the early stages of scabies, and the antibody could be used for detection throughout infection. As well as infestation, PTK may play an important role in the immune response. Three out of nine serum samples from *P. ovis cuniculi*-infested rabbits showed cross-reactivity, possibly because scabies mites share a close evolutionary relationship with *P. ovis cuniculi* mites. Nevertheless, cross-reactivity experiments confirmed the ability to distinguish mite infestation from other parasitic infestations in rabbits. It is noteworthy that total IgG levels varied during the infestation process, with a highly significant difference between naïve and *Eimeria* spp. infestation, *C. pisiformis* infestation and *P. ovis cuniculi* infestation groups (p < 0.01). In particular, total IgG levels in sera from the *P. ovis cuniculi* infestation group were significantly higher than those in the *Eimeria* spp. group (p > 0.01) and *C. pisiformis* infestation groups (p < 0.05), indicating that there was no cross-reactivity when using serum samples from rabbits infested with *Eimeria* spp. or *C. pisiformis*. Taken together, the results demonstrate that recombinant SsPTK has potential as a sensitive antigen for the diagnosis of parasitic mites during the early stages of infection. These findings expand our understanding of rabbit immune responses during infestation and further support the development of recombinant PTK as a serodiagnostic agent for detecting *S. scabiei* during the early stages of infestation.

## Methods

### Ethics statement

All rabbits were handled in strict accordance with the animal protection laws of the People’s Republic of China (draft released on September 18, 2009). All procedures were implemented in strict accordance with the Guide for the Care and Use of Laboratory Animals of the Animal Ethics Committee of Sichuan Agricultural University (Ya’an, China; Approval No. 2013-028). All methods were carried out in accordance with relevant guidelines and regulations, including any relevant details.

### Source and sera

A total of 42 New Zealand White rabbits naturally infested with *S. scabiei* for two months were provided by the Department of Parasitology, College of Veterinary Medicine, Sichuan Agricultural University. Mites were stored in liquid nitrogen after collection from infested rabbits. RNA isolation from mites as performed with an RNA isolation kit (Waston, Shanghai, China) and RNA was transcribed into cDNA using a cDNA synthesis kit (Thermo, Shanghai, China) and stored at −70 °C until needed. Total crude protein was extracted from mites using a total protein extraction kit (BestBio, Shanghai, China). Serum samples (42) were collected from rabbits displaying itching, thick scabs on skin, and evidence of mites in skin scrapings under a microscope^2, 34^ that were assumed to be infected with *S. scabiei*. Blood samples from 24 New Zealand White rabbits without itching, skin scabs or evidence of mites were collected as negative controls to determine the assay cut-off value and to perform cross-reactivity tests. Serum samples from rabbits naturally infested with *Eimeria* spp. (nine samples), *Psoroptes ovis cuniculi* (nine samples), and *Cysticercosis pisiformis* (nine samples) were collected from three rabbit farms in Sichuan Province. Serum samples were also collected from another 24 healthy rabbits before artificial infestation as controls, and from the same 24 rabbits after artificial infestation with mites for 1 or 2 weeks. All blood samples were stored at −20 °C until needed. All rabbits were from rabbit farms in Sichuan Province, China.

### Cloning, expression and purification of SsPTK

After identifying a full-length PTK cDNA from *S. scabiei* transcriptome data (GenBank: KY080515), the ORF encoding SsPTK was amplified. Primers (Invitrogen, Beijing, China) were designed using Primer 5.0 software and were as follows: forward, 5′-CGG GAT CCA TGC TCA AAA GTT ACG CTG TTC-3′, with a *BamH*I restriction site (underlined); reverse, 5′-CGA GCT CTT ATG TTA TCG TAG ATG TTG TTG CT-3′, with a *Sac*I restriction site (underlined). *SsPTK* was amplified by PCR with cycling conditions of 94 °C for 5 min, followed by 35 cycles of amplification at 94 °C for 45 s, 62 °C for 45 s, and 72 °C for 45 s, and a final extension at 72 °C for 10 min. The resulting fragment was cloned into the pET32a(+) expression vector (Invitrogen, Beijing, China), and the construct was transformed into *Escherichia coli* BL21 (DE3) (TIANGEN, Beijing, China) for protein expression which was induced with 1 mM isopropyl-β-D-thiogalactoside (IPTG) at 37 °C for 10 h. Recombinant SsPTK protein was purified from inclusion bodies in denaturing conditions (8 M urea) by chromatography using a Ni-NTA His-tag affinity kit (Bio-Rad, USA) according to the manufacturer’s instructions.

### Sequence analysis

We obtained the complete *S*sPTK sequence and translated the amino acid sequence using DNAStar software (version 7.0), and DNAMAN (version 7.0) was employed to compare similarity between homologous genes. SsPTK was analysed using SignalP 4.1 (http://www.cbs.dtu.dk/services/SignalP/), Transmembrane Prediction Server (http://www.sbc.su.se/~miklos/DAS/), TargetP (http://www.cbs.dtu.dk/services/TargetP/) and ExPasy (http://web.expasy.org/protparam/) to respectively assess potential signal peptides, transmembrane regions and subcellular localisation, and to calculate the predicted molecular weight and pI values. Homologous proteins were found in the NCBI database and comparative analysis was performed using the online software Clustal W2 (http://www.ebi.ac.uk/tools/msa/clustalw2/). Finally, we used MEGA 5.05 software to construct an evolutionary tree with 1000 bootstrap replicates by the neighbour-joining (NJ) method with sequences from 24 species. We employed Poisson correction to account for gaps and missing data.

### Western blotting analysis

Samples (40 μL of protein and 10 μL loading buffer) were boiled for 10 min, separated by 12% SDS-PAGE, and transferred to a nitrocellulose membrane over 35 min using a trans-blot SD semi-dry transfer cell (Bio-Rad, USA) at room temperature. Membranes were washed three times for 5 min each time in Tris-buffered saline-Tween- (TBST) (20 mM TRIS-HCl, 150 mM NaCl, 0.05% v/v tween-20, pH 7.4), coated in 5% skimmed milk for 2 h, then incubated overnight with positive serum (diluted 1:100 with 0.01 M PBS). Next, membranes were washed four times for 5 min each time in TBST, then incubated with horseradish peroxidase (HRP)-conjugated goat anti-rabbit antibody (Earthox, USA) (diluted 1:1000) for 2 h. Membranes were washed four more times, and signals were detected using diaminobenzidine reagent (TIANGEN, Beijing, China).

### Preparation of recombinant SsPTK polyclonal antibody

Two male New Zealand White rabbits were included in this experiment. Rabbit negative sera were collected before immunisation. Rabbits were immunised three times with 7 days between the first and second immunisations and 14 days between the second and third. The first immunisation was with 200 μg of purified recombinant SsPTK emulsified with an equal volume of Freund’s complete adjuvant (Sigma, USA), and subsequent immunisations were with 100 μg of purified recombinant protein and the same volume of Freund’s incomplete adjuvant (Sigma, USA). Antiserum samples were collected after 2 weeks and used to test the antibody titre. Antiserum was purified using a Protein G-Sepharose column (Bio-Rad, USA) according to the manufacturer’s instructions to separate immunoglobulin G (IgG; specific rabbit anti-PTK antibodies) from other serum components.

### Immunofluorescence

Adult mites were fixed in 1% molten agarose a short time after collection, and solid agarose containing the mites was embedded in paraffin wax and cut into thin sections (5 μm) with a microtome. The sections were baked in a 60 °C oven for 2 h, dewaxed in xylene twice for 7 min each time, in 100% ethanol twice for 3 min each time, in 95% ethanol for 3 min, in 85% ethanol for 3 min, in 75% ethanol for 3 min, and rinsed with distilled water for 8 min. To inactivate endogenous peroxidase, sections were incubated in blocking buffer (3% H_2_O_2_ in PBS) for 20 min at 37 °C. Heat-induced epitope retrieval was accomplished by immersing sections in 0.01 M sodium citrate buffer (pH 6.0) at 95 °C for 20 min. Sections were incubated in blocking buffer (5% bovine serum albumin in PBS) for 1 h at room temperature before incubation overnight at 4 °C with specific rabbit anti-PTK antibodies (diluted 1:100 in PBS). After washing three times with PBS, sections were incubated with fluorescein isothiocyanate goat anti-rabbit IgG (H+L; Amresco, Texas, USA) diluted 1:100 in 0.1% Evans Blue for 1 h at 37 °C in the dark. Finally, sections were viewed with a microscope. In this experiment, negative controls were naïve rabbit serum instead of specific antibodies.

### Development of indirect ELISA and detection of serum samples

A checkerboard titration study was carried out to determine the optimal conditions for recombinant SsPTK protein and serum^42^. ELISA procedures were as follows: firstly, 96-well culture plates were coated with 100 μL of protein diluted in 0.1 M carbonate buffer (pH 9.6) and incubated overnight at 4 °C. Secondly, plates were washed with PBS-T (137 mM NaCl, 2.7 mM KCl, 10 mM Na_2_HPO_4_, 2 mM KH_2_PO_4_, 0.1% v/v tween-20, pH 7.4) three times for 5 min each time, then incubated with blocking buffer (5% skimmed milk diluted in PBS) at 37 °C for 90 min. Thirdly, after washing the plates with PBS-T three times for 5 min each time, serum samples were diluted 1:80 with PBS, and 100 μL was added to each well and plates were incubated at 37 °C for 1 h. Plates were washed as before, then secondary antibody (100 μL goat anti-rabbit IgG-horseradish peroxidase antibody diluted 1:5000 with PBS) (Earthox, USA) was added to each well and incubated at 37 °C for 1 h. Plates were washed as before and colour development was performed by adding the substrate 3,3,5,5-tetramethylbenzidine (TIANGEN, Beijing, China) to each well (100 μL) for 15 min. The reaction was stopped by the addition of 100 μL of 2 M H_2_SO_4_ to each well and the optical density (OD) was determined at 450 nm using an ELISA plate reader.

In optimal conditions, 24 serum samples from naïve rabbits free from *S. scabiei* were tested to determine the cut-off value for indirect ELISA. The cut-off value serves as an identity standard for negative and positive serum, and was calculated as the arithmetic mean of the OD_450_ values from negative serum samples plus three standard deviations^43, 44^. Serum samples from rabbits infested with *S. scabiei* (42 in total) were tested to detect anti-PTK antibody. Cross-reactivity tests were undertaken to determine the antigen specificity using serum samples from naïve rabbits (n = 24) and from rabbits infested with three other common parasites: *Eimeria* spp. (n = 9), *P. ovis cuniculi* (n = 9) and *C. Pisiformis* (n = 9). Serum samples from 24 healthy rabbits before artificial infestation served as the control, and samples were collected from the same rabbits after artificial infestation with mites for 1 and 2 weeks to assess the performance of PTK in early diagnostic tests. We determined the optimal dilutions of the PTK antigen and rabbit serum that gave rise to the maximum difference in OD450 nm values between positive and negative sera (P/N)^45^. Negative, positive and blank controls were included on each plate.

### Repeatability and reproducibility of SsPTK ELISA

To evaluate the repeatability and reproducibility of the PTK ELISA, six positive serum samples were used. Every sample was tested simultaneously to assess the intra-assay variability (repeatability), and then tested consecutively to assess the inter-assay variability (reproducibility). Repeatability and reproducibility tests were conducted three times and their corresponding mean OD_450_ values, standard deviation (SD), and coefficient of variation (CV) were calculated.

### Early diagnosis testing of SsPTK ELISA

We assessed the early serodiagnostic potential of the recombinant SsPTK-based ELISA. Serum samples from 24 healthy rabbits were collected before artificial infestation as controls, and from the same rabbits after artificial infestation with mites for 1 and 2 weeks to assess the early diagnostic value SsPTK. The positive diagnostic rate was calculated by referring to the cut-off value. Each rabbit was tested three times.

### Data analysis

All analysis was carried out using GraphPad Prism version 5.0 (GraphPad Software). The significance of differences between groups was accomplished using IBM SPASS statistics 2.0 (SPASS Software). As a measure of potential diagnostic performance, sensitivity and specificity were calculated for ELISA. Cut-off was calculated as the arithmetic mean of the OD_450_ values from negative serum samples plus three standard deviations. Sensitivity percentage was calculated using the formula: ELISA positive/scabies positive rabbit ×100, where the specificity percentage was calculated from: ELISA negative/scabies negative rabbit ×100^21^.

## Electronic supplementary material

Supplementary Information
