# Supplementary material for: Expression and characterisation of a Sarcoptes scabiei protein tyrosine kinase as a potential antigen for scabies diagnosis
Source: Sci Rep. 2017 Aug 29;7:9639. doi: 10.1038/s41598-017-10326-w (PMC5575040; doi:10.1038/s41598-017-10326-w)
Supplement: Supplementary file 1 — Supplementary Information [file 41598_2017_10326_MOESM1_ESM.pdf]

**Supplementary Information**

# Expression and characterisation of a *Sarcoptes scabiei* protein tyrosine kinase as a potential antigen for scabies diagnosis

Nengxing Shen[1+], Ran He[1+], Yuqing Liang[1], Jing Xu[1], Manli He[1], Yongjun Ren[2,3], Xiaobin Gu[1], Weimin Lai[1], Yue Xie[1], Xuerong Peng[4], Guangyou Yang[1*]

[1] Department of Parasitology, College of Veterinary Medicine, Sichuan Agricultural University, Wenjiang, 611130, China

[2] Sichuan Animal Sciences Academy, Sichuan Chengdu, 610066, China

[3] Animal Breeding and Genetics key Laboratory of Sichuan Province, Sichuan Chengdu, 610066, China

[4] Department of Chemistry, College of Life and Basic Science, Sichuan Agricultural University, Wenjiang, 611130, China

[*]Corresponding author

E-mail: guangyou1963@aliyun.com

[+] These authors contributed equally to this work.

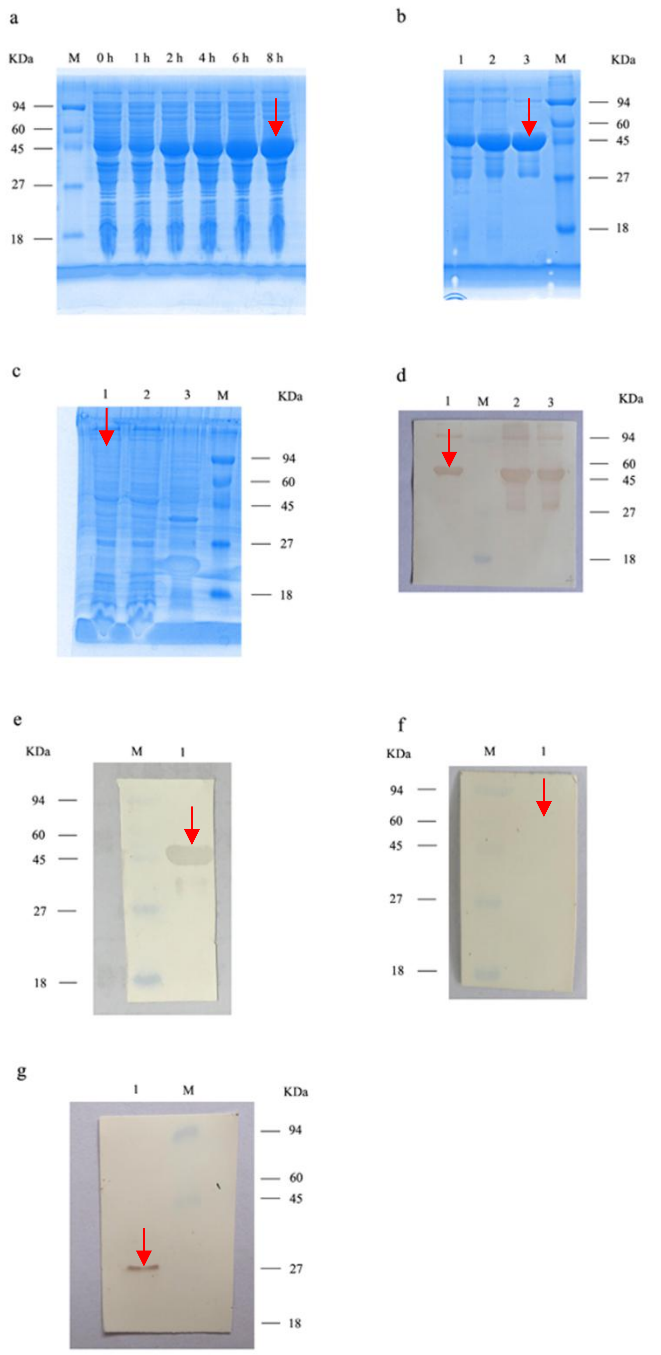


Figure S1 Full-length SDS-PAGE and western blot analysis of *S. scabiei* PTK. (a) SDS-PAGE of *S. scabiei* PTK under different induction time. Lanes are as follows: lane M, protein molecular weight markers (in kDa); lane o h, 1 h, 2 h, 4 h, 6 h and 8 h, induction for o h, 1 h, 2 h, 4 h, 6 h and 8 h, respectively. (b) SDS-PAGE of *S. scabiei* purified PTK. Lanes are as follows: lane M, protein molecular weight markers (in kDa); lane 1: 100 mM elution concentration of imidazole; lane 2: 150 mM elution concentration of imidazole; lane 3: 200 mM elution concentration of imidazole. (c) SDS-PAGE of *S. scabiei* total crude protein. Lanes are as follows: lane M, protein molecular weight markers (in kDa); lane 1: total crude protein; lane 2: total crude protein as parallel control; lane 3: pET32a (+) vector protein. (d) Western blot of different volume purified rPTK detection with serum from a rabbit naturally infested with *S. scabiei*. Lanes are as follows:

lane M, protein molecular weight markers (in KDa); lane 1, lane 2 and lane 3: 30 μg, 50 μg and 40 μg purified rPTK detection with serum from a rabbit naturally infested with *S. scabiei*, respectively. (e) Western blot of purified rPTK detection with rabbit anti-PTK serum. Lanes are as follows: lane M, protein molecular weight markers (in KDa); lane 1, 30 μg purified rPTK detection with rabbit anti-PTK serum. (f) Western blot of purified rPTK detection with naïve rabbit serum. Lanes are as follows: lane M, protein molecular weight markers (in KDa); lane 1, 30 μg purified rPTK detection with naïve rabbit serum. (g) Western blot of total crude proteins detection with rabbit anti-PTK serum. Lanes are as follows: lane M, protein molecular weight markers (in KDa); lane 1, 30 μg total crude proteins detection with rabbit anti-PTK serum.

Table S1 Determination of the coating concentration of protein and serum dilution in optimization of the indirect ELISA method

| Antisera at different dilutions | OD450 values of antigen at different coating concentrations | | | | | | |
|---|---|---|---|---|---|---|---|
| | 32 μg/mL | 16 μg/mL | 8 μg/mL | 4 μg/mL | 2 μg/mL | 1 μg/mL | 0.5 μg/mL |
| 1:10 (P) | 1.201 | 1.074 | 1.109 | 1.110 | 1.053 | 0.993 | 0.914 |
| 1:10 (N) | 0.243 | 0.227 | 0.211 | 0.203 | 0.199 | 0.172 | 0.148 |
| 1:20 (P) | 1.126 | 1.054 | 1.068 | 1.042 | 1.033 | 0.971 | 0.897 |
| 1:20 (N) | 0.227 | 0.214 | 0.190 | 0.163 | 0.155 | 0.150 | 0.132 |
| 1:40 (P) | 1.128 | 1.106 | 1.030 | 1.037 | 0.969 | 0.909 | 0.868 |
| 1:40 (N) | 0.211 | 0.192 | 0.177 | 0.164 | 0.152 | 0.139 | 0.118 |
| 1:80 (P) | 1.058 | 1.045 | 1.037 | **1.014** | 0.953 | 0.873 | 0.851 |
| 1:80 (N) | 0.181 | 0.172 | 0.154 | **0.127** | 0.130 | 0.122 | 0.119 |
| 1:160 (P) | 0.993 | 0.971 | 0.950 | 0.909 | 0.874 | 0.847 | 0.820 |
| 1:160 (N) | 0.167 | 0.152 | 0.147 | 0.129 | 0.123 | 0.119 | 0.108 |
| 1:320 (P) | 0.914 | 0.894 | 0.832 | 0.811 | 0.776 | 0.750 | 0.722 |
| 1:320 (N) | 0.149 | 0.143 | 0.131 | 0.126 | 0.118 | 0.117 | 0.107 |

Note: P, positive serum; N, negative serum; the values in bold represent optimum conditions of this indirect ELISA method.
